# Experimental test and mechanism analysis of soil crust erosion resistance of rammed earth Great Wall in rainy season

**DOI:** 10.1038/s41598-024-59706-z

**Published:** 2024-04-20

**Authors:** Liang Liu, Yun Zhang, Lianjun Guo, Haiying Cao, Zhenwei Dai, Zhiyong Zhao, Ying Guo, Dongdong Li, Lingling Zheng, Tianli Li

**Affiliations:** 1 School of Earth Sciences and Engineering, Nanjing University, Nanjing, 210023 China; 2 The Eighth Geological Brigade, Hebei Bureau of Geology and Mineral Resources Exploration, Qinhuangdao, 066001 China; 3 School of Civil Engineering and Mechanics, Yanshan University, Qinhuangdao, 066004 China; 4 Wuhan Center, China Geological Survey (Central South China Innovation Center for Geosciences), Wuhan, 430205 China; 5 Cultural Tourism Bureau, Shanhaiguan, Qinhuangdao, 066200 China; 6 Hebei Jianyan Architectural Design Co. Ltd., Shijiazhuang, 050000 China; 7 National Aquatic Biological Resource Center, Institute of Hydrobiology, Chinese Academy of Sciences, Wuhan, 430072 China

**Keywords:** Rammed earth great wall, Erosion resistance, Genome sequencing, Biological soil crust, Civil engineering, Soil microbiology, Natural hazards

## Abstract

Rammed earth is a kind of cleaning material, widely used in all kinds of buildings in the world. The Great Wall of ancient China is a typical world cultural site built from rammed earth. The rammed earth Great Wall of Shanhaiguan is close to Bohai Bay, which has suffered from long-term erosion by rain, causing a series of problems such as soil loss, collapse and gully flushing. The protection materials of the rammed earth site have always puzzled scholars. However, during the rainy season, it was found that some of the walls at Xiaowan Gouge and Nantuzhuang Gouge in the Shanhaiguan Great Wall had unwashed traces, the soil surface of the walls was intact, and the anti-erosion ability of the walls was significantly higher than that of other places. In order to explore the reasons for its strong anti-erosion ability in the natural state of rammed earth wall, guide the protection of rammed earth Great Wall, and carry out different experimental tests to explore its anti-erosion reasons and internal mechanisms. Firstly, the characteristics of rammed soil were understood through the composition test of rammed soil, and the indoor and outdoor erosion test was carried out to determine that the anti-erosion reason was the protection of gray-green soil crust. The property and composition of soil crust were determined through the immersion test and genome sequencing. Finally, the protection mechanism of soil crust was analyzed by scanning electron microscopy.

## Introduction

Rammed earth buildings are buildings formed by the artificial ramming of natural soil. Rammed earth is a renewable and clean energy raw material^1,2^ that is able to meet the current carbon emission requirements^3–5^. It is widely used in various fields in the world, such as the Great Mosque in Mali, the earth cellar on Canada's main island, and the great wall of China^6,7^. The Great Wall is an important military defense system of China and a world historical and Cultural Heritage^8,9^. The Great Wall of Shanhaiguan was built during the Ming Dynasty. The wall is mainly composed of bricks externally and rammed earth internally. After hundreds of years of extreme weather conditions, the bricks of some sections of the Great Wall fell away, leaving only the rammed earth walls. Under the action of long-term rainwater erosion, wall erosion and collapse caused serious and irreversible damage to historical relics. Therefore, it is necessary to pay attention to the erosion resistance of the rammed earth Great Wall.

In the past, curing agents were often used in the restoration of rammed earth sites^10^. Biological enzyme curing agents, such as microbially induced calcite precipitation (MICP) technology^11–13^. An example of an inorganic curing agent is potassium silicate with a high modulus (PS)^14–16^. PS is diluted with water to form silicate hydrate, the silicate anions and the metal cations in the clay minerals are electrostatically adsorbed, and the sheet clay minerals are connected to form aluminosilicate gels. An example of an organic curing agent is modified polyvinyl alcohol (SH), which is a high molecular polymer that reacts with soil to produce hydrocarbons and other derivatives, resulting in ion exchange and flocculation that cement soil particles together^17,18^. All the above are manual soil surface treatment to improve soil anti-erosion performance. However, after investigation, no manual treatment has been carried out here, and the local erosion resistance of this place is naturally improved. The discovery that the protection of natural materials has better adaptability than traditional restoration methods has aroused our great interest. We first need to determine what this grayish-green thin layer of material is that forms under natural conditions. as well as its resistance to erosion, and reveal its mechanism. so that it can be applied to the conservation of rammed earth sites.

Soil crust is a natural phenomenon that exists widely on the soil surface in arid desert areas all over the world^19,20^. Soil crust can be divided into biological soil crust (BSC)^21,22^ and physical soil crust (PSC) (J. et al., 1984; Mcintyre, 1958^23,24^ according to its morphology and genesis. BSC are mainly composed of cyanobacteria, lichens and algae, as well as bacteria, mosses and fungi^25^. PSC usually forms through the consolidation of soil materials due to rain effects or through the redistribution and accumulation of fine particles during surface runoff^24,26,27^. Soil crust can significantly affect ecosystem processes^28^, Including desert system ecology^29–31^, soil hydrology^32^, soil physicochemical properties^33,34^ and the carbon cycle^35^. Previous studies have found that biological soil crust occurs mostly in arid and semi-arid areas, and rarely in areas with abundant rainy season. At the same time, the soil properties of the place are mainly sandy soil, and less appear in the cohesive soil of rammed earth. Therefore, we need to identify the soil crust characteristics of the gray-green thin layer structure found at the rammed earth site in Bohai Bay.

In order to explore the reasons for the high local anti-erosion ability of the rammed earth Great Wall in Shanhaishan during the rainy season, different testing methods were adopted to explore its formation reasons and internal mechanism. Firstly, the characteristics of rammed earth in the Great Wall are tested. Then through the field erosion test and indoor erosion test, it is confirmed that the gray-green soil crust on the surface of rammed soil improves the erosion resistance of the soil. After that, the biological characteristics of soil crust were determined by immersion test and genome test. Finally, the internal mechanism of soil crust resistance to erosion was analyzed. The research results will provide important guidance for the protection and restoration of the Great Wall site, avoid the disaster of erosion and erosion, and provide promoting significance for the construction of the Great Wall cultural park in China.

## Geological setting and history of the Great Wall

### Physical geography

**Figure 1:**
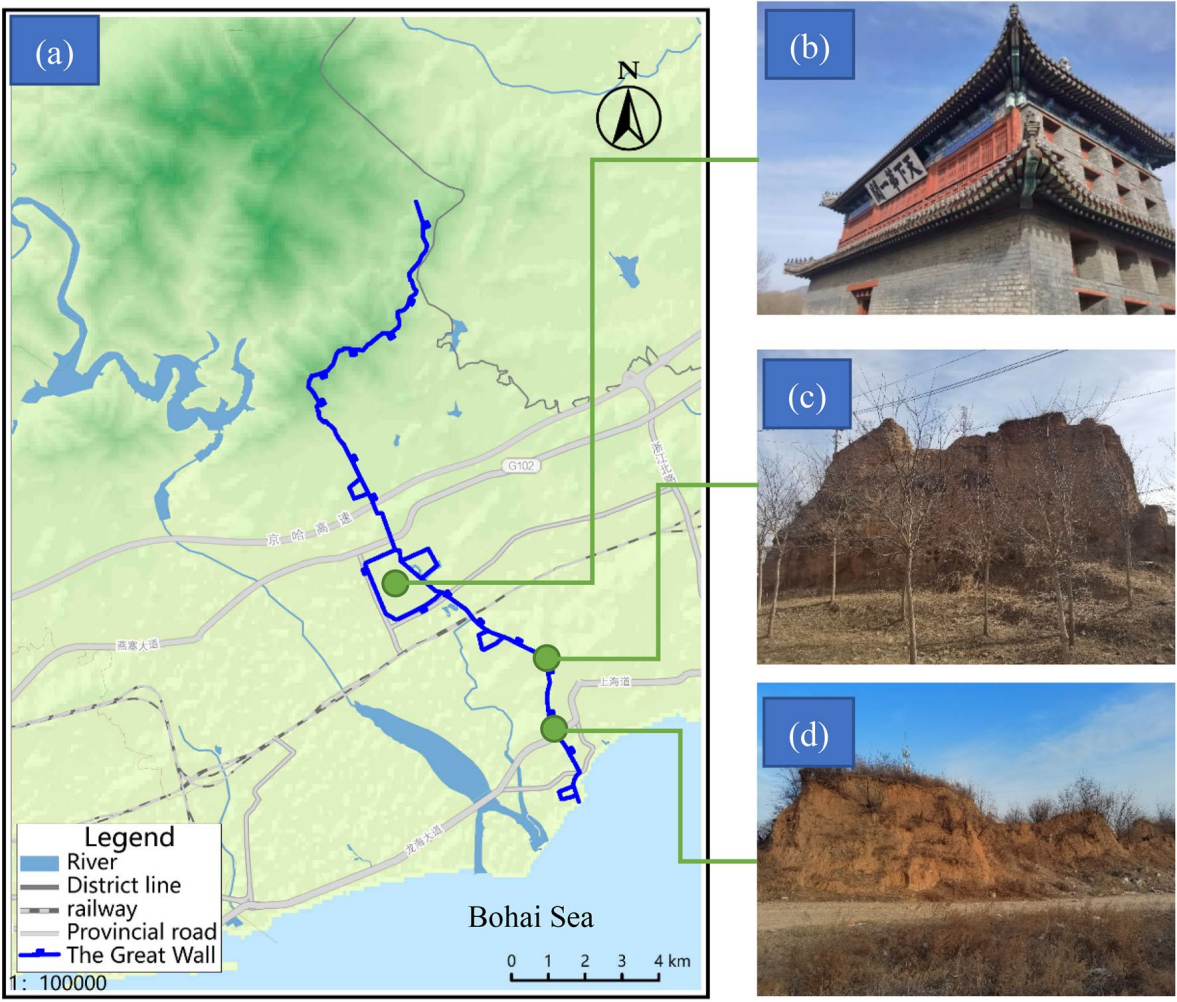
The Great Wall of Shanhaiguan, China: (**a**) Shanhaiguan Great Wall location map (This figure were generated used by the software Bigemap, the URL link is http://www.bigemap.com/), (**b**) the first pass in the world, (**c**) Nantuzhuang Great Wall gap, (**d**) Xiaowan Great Wall gap.

The Shanhaiguan Great Wall is located in Shanhaiguan District, Qinhuangdao City, Hebei Province, China (Fig. 1). It was built in the 14th year of Hongwu during the Ming Dynasty (AD 1381). As an important military defense system in ancient China against northern aggression, it was deemed a World Cultural Heritage site in December 1987^8^.

The Great Wall of Shanhaiguan is adjacent to Bohai Bay and experiences a temperate continental monsoon climate with four distinct seasons. According to the data of the Qinhuangdao Meteorological Bureau, the average annual temperature of Qinhuangdao is 10.5 °C, the highest monthly average temperature, 24.4–25.3 °C, occurs in July, and the lowest monthly average temperature, -8.4 to -4.7 °C, occurs in January. The extreme maximum temperature in the area is 40.3 °C, and the extreme minimum temperature is -29.2 °C. The average annual precipitation is 645.3–693.5 mm, the maximum annual precipitation is 1273.5 mm, and the annual minimum precipitation is 332 mm. Precipitation is mostly concentrated from June to August, with an average precipitation of 399.9–460.6 mm, accounting for 68% of the average annual precipitation. From November to February, the precipitation is the lowest, with an average precipitation of 18.78 mm, accounting for 3.18% of the average annual precipitation. The maximum daily rainfall is 291.0 mm (1984.8.10), and the maximum accumulated rainfall is 614 mm (July 20, 2016 4:00 a.m.—July 21, 2016 8:00 a.m.). The annual number of sunshine hours ranges from 2403–3113 h.

### History of the Great Wall of Shanhaiguan, China

**Figure 2:**
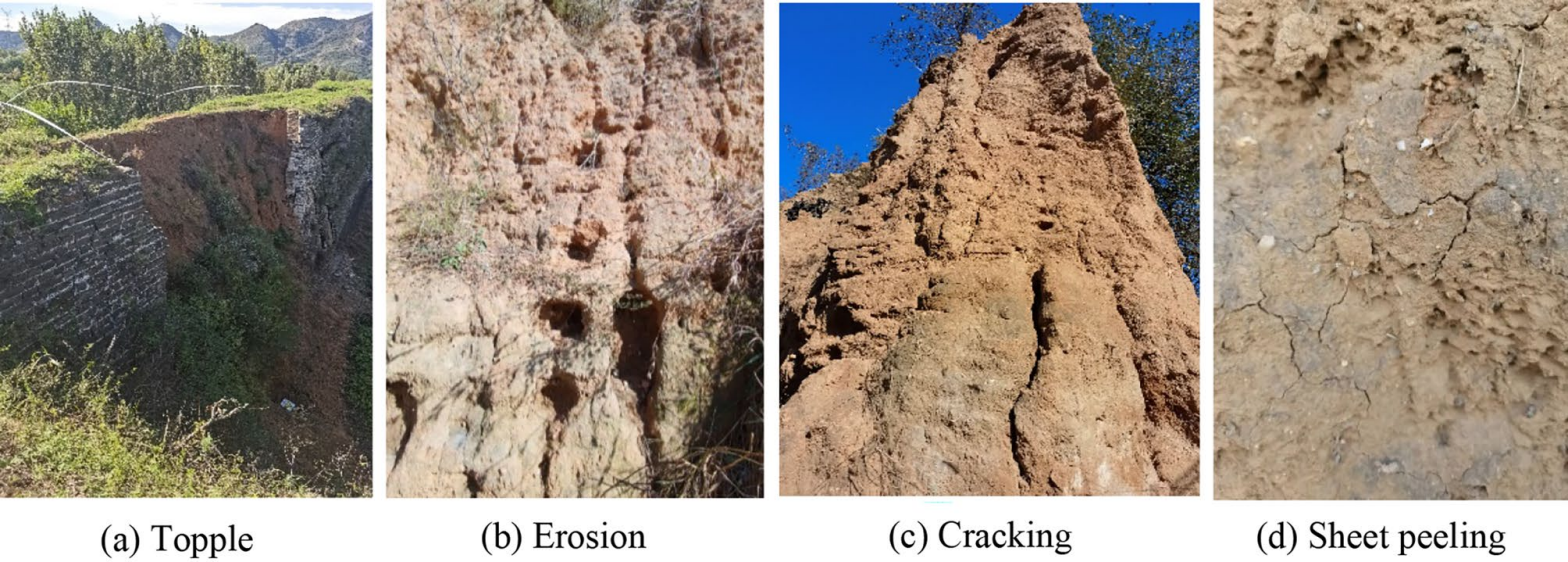
Types of damage to the Great Wall.

Since the Great Wall was built a long time ago, it has suffered various degrees of damage due to natural, war and man-made effects over hundreds of years. According to historical records, the Great Wall of Shanhaiguan has been renovated many times in history; it was repaired during the Qing Dynasty, and it was repaired many times after the founding of New China. The foundation of the Great Wall of Shanhaiguan adopts a strip stone foundation, the surface of the wall is covered with sintered gray bricks, and the inner wall core is rammed earth. The cross-section of the Great Wall is trapezoidal, the wall height is approximately 8 m, the top width is approximately 3–5 m, and the bottom width is approximately 6–8 m (Fig. 1c). In some areas, the wall tiles on both sides have fallen off, leaving only the rammed earth wall core. The rammed earth wall core is mainly silty clay, with gravel in some sections. After the rammed earth wall core loses the protection of the wall tiles, the soil is eroded by the rainwater, forming a gulley. After sun exposure, the wet wall dries, and the water evaporates, resulting in dry cracks. Repeated rainfall and sun exposure results in shrinkage–expansion cycles and salt deposition for the rammed earth wall^10^. This leads to a series of problems, such as soil deterioration and collapse (Fig. 2).

### Characteristics of the local erosion resistance of the rammed earth wall

Rammed earth has unique properties as the core of the Great Wall. According to historical records, the rammed earth core of the Great Wall was built together with the wall bricks during the masonry process of the Great Wall. The soil was made of silty clay mixed with gravel^36^. Using a rammer, multiple people lifted and then dropped it to ram the soil to form a dense structure. Some reinforced sections were mixed with lime, glutinous rice pulp, etc., to enhance the strength of the wall. Soil materials were often obtained from nearby areas to facilitate their use. The rammed wall core soil has greater strength and can stand upright even if the protection of the wall tiles is lost.

**Figure 3:**
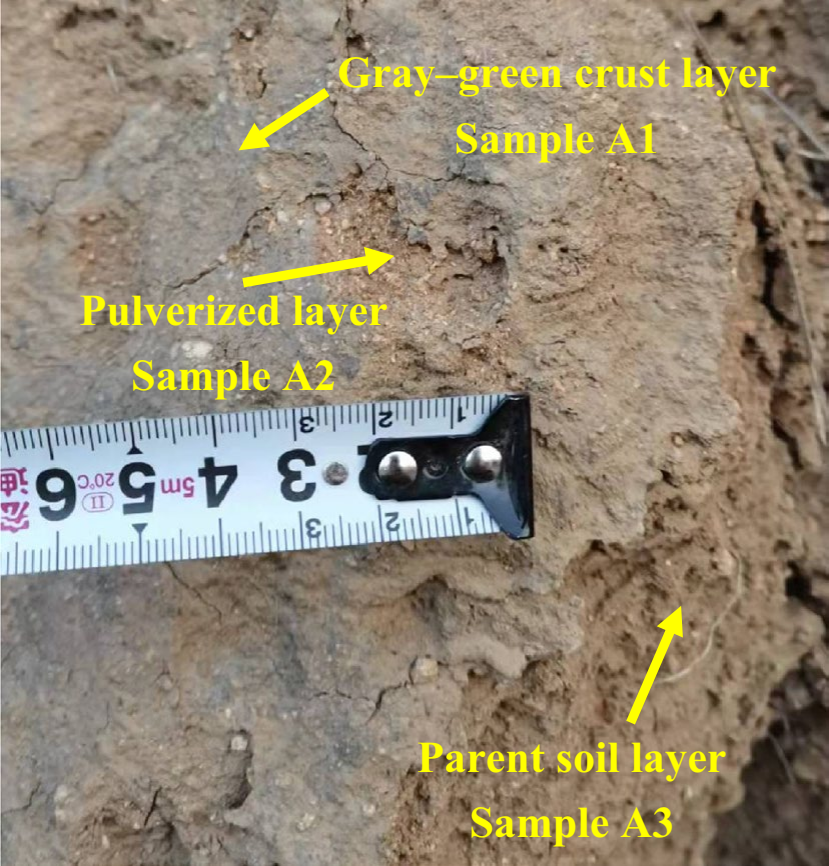
Features of the Xiaowan Gap of the Great Wall.

Field observations show that the Xiaowan Gap and Nantuzhuang Gap of the Great Wall have the same characteristics as the erosion resistance wall parts; that is, a gray‒green thin-layer structure is formed on and covered the soil surface. The structural layer is relatively dense, and the thin-layer structure in some sections can be peeled off by prying it with a knife. The thickness of the thin-layer soil skin measured with a Vernier caliper is approximately 1–2.5 mm. Previous researchers called this thin-layer soil skin the crust layer^23,37–40^. After peeling off, the second layer of soil particles is large, rough, loose in structure, easily falls off under external force and is a pulverized layer. The second layer can be scraped off with a knife. The soil no longer falls off in powder form, the soil structure is compact, and the particle gradation is relatively uniform. It is the original structure of rammed earth and is the parent soil layer (Fig. 3).

## Materials and methods

### Rammed earth materials

The soil samples used in this experiment were obtained from the Great Wall of Shanhaiguan. The rammed earth Great Wall surface crust layer is sample A1 (gray‒green thin-layer structure), the powder layer of the lower crust layer is sample A2, the wall parent soil is sample A3, the weathered rock block near the wall is sample A4, the rammed earth from the parent soil is sample A5, Sample sampling locations are shown in Table 1. Powder B1 and B2 made from A5 rammed soil sample. Six to ten pieces of each sample were taken for parallel testing.

**Table 1:** Test samples.

Sample number	Sampling location	Sample picture
A1	The grey-green thin layer structure on the surface of Shanhaiguan rammed earth Great Wall	
A2	Obtained by scraping the A1 layer with a knife	
A3	Sample of the interior of a rammed earth wall in its original state	
A4	Rock samples around the Great Wall of rammed earth	
A5	The rammed earth from the parent soil	

The process of A5 sample fabrication is as follows: the mother soil sample of the wall taken was dried and processed, and the dried soil sample was crushed with a wooden hammer and passed through a 2 mm sieve. A homemade 7 × 7 × 7 cm mold was used as the compacted sample mold. According to the measured optimal moisture content of water, according to 16% moisture content of water with a spray bottle, evenly sprayed on the surface of the soil body, into a sealed bag, sealed for 24 h standby. The weight of 2.5 kg tamping hammer was used to tamp in three layers, each layer was filled with 213 g of soil, and each layer was tamped with 5 strikes, and finally compacted to a uniform height with 2000psi pressure under a hydraulic press. The size of the specimen was 7 × 7 × 6.7 cm, weighing 639 g, and the specimen was cured under natural condition for 28 days after the specimen was made.

### Composition and structural characteristics of rammed earth

#### Rammed earth Great Wall parent soil composition

The parent soil of the A3 sample rammed earth Great Wall is silty clay, which is yellowish-brown. The soil texture is hard. The test method is in accordance with the requirements of "Standards for Geotechnical Test Methods" (GB/T50123-2019). Its physical and mechanical properties are shown in Table 2.

**Table 2:** Physical and mechanical property parameters of the A3 sample rammed earth.

Specimen	ω%	*ρ* (g/cm^3^)	G_s_	S_r_ %	ω_L_ %	ω_P_ %	I_P_	ω_op_%
A3	13.5	2.01	2.74	67.6	35.3	19.1	16.2	15.4

ω denotes the water content; *ρ* is the density; G_s_ represents the specific gravity; S_r_ is the saturation; ω_L_ and ω_P_ are the liquid limit and plastic limit, respectively; I_P_ corresponds to the plasticity index; ω_op_ is the optimum water content.

**Figure 4:**
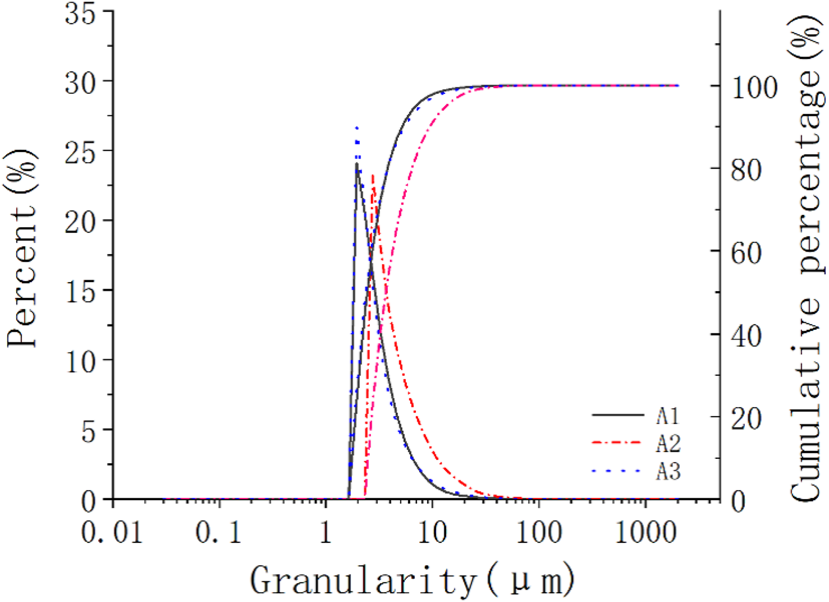
Particle analysis curve.

The fine particle content of samples A1, A2 and A3 was tested, and the results were shown in Fig. 4. In the figure, the left side is the percentage of each grain group, and the right side is the cumulative percentage. As can be seen from Fig. 4, the content of clay particles in the layer A1 is the highest, followed by A3 in the parent soil layer, and A2 in the silt layer is the least. The silt content in the silted layer A2 is the highest, followed by the mother soil layer A3, and the layer A1 is the least.

#### Identification of glutinous rice syrup and lime mixture

**Figure 5:**
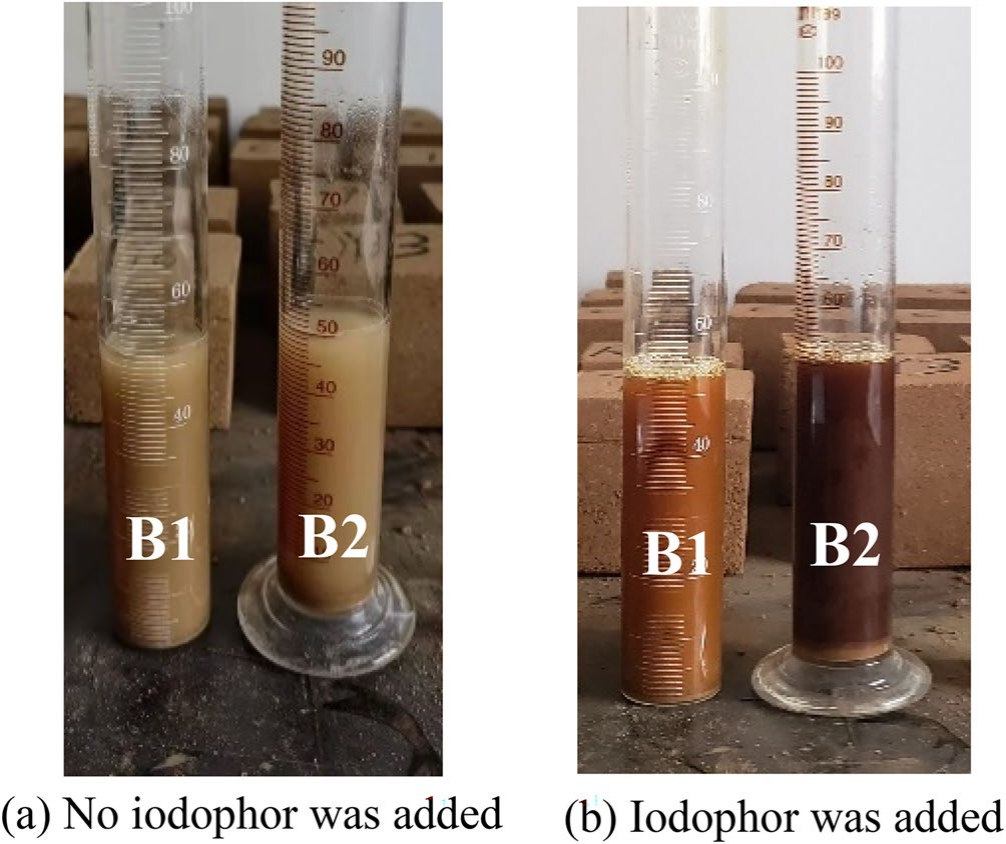
Starch titration test photo.

According to historical records, glutinous rice pulp and lime were added to some sections of the Great Wall of Shanhaiguan during the construction period. In this way, the mechanical strength of the rammed earth Great Wall was increased, coupled with the protection of the outer brick to improve the overall performance of the Great Wall. The main component of glutinous rice pulp is starch. By adding iodine to the rammed earth aqueous solution, it is determined whether glutinous rice pulp was incorporated. The test method is shown in Table 3. From the comparison of Fig. 5a and b, it can be seen that the B1 specimen did not show a color reaction, while the B2 specimen did show a color reaction after artificially adding glutinous rice slurry into the rammed soil.

**Table 3:** Starch titration test.

Specimen number	Experimental test method
B1	Mix 1 g rammed earth powder sample, 50 ml deionized water, and 3 ml iodophor, test 5 groups of parallel experiments
B2	Mix 1 g rammed earth powder sample, 50 ml deionized water, 2 ml glutinous rice syrup, and 3 ml iodophor, test 5 groups of parallel experiments

Under long-term action, CaCO_3_ will be produced if lime is added to the rammed earth in the wall. Diluted hydrochloric acid with a concentration of 0.5 mol/L was added to crust layer sample A1, pulverized layer sample A2, parent soil layer A3, and no foaming occurred.

**Figure 6:**
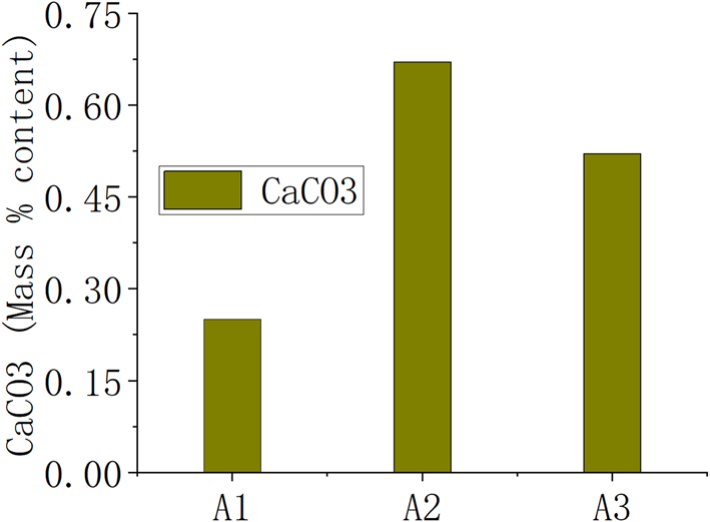
CaCO_3_ mass % in soil.

The CaCO_3_ content of A1, A2 and A3 samples was identified. The test method was carried out in accordance with the requirements of the Standard for Geotechnical Test Methods (GB/T50123-2019), and the results are shown in Fig. 5. It can be seen from Fig. 6 that the CaCO_3_ contents of the three samples of A1, A2, and A3 are low, with the mass percent contents of 0.25%, 0.67%, and 0.52%.

According to the ancient literature and a large amount of archaeological data, the formula of the rammed earth of the Great Wall at Shanhaiguan does contain ingredients such as glutinous rice paste and lime. However, after a long time of weathering, chemical action and decomposition, not much of the original ingredients such as glutinous rice paste and lime are left. Through calcium carbonate content testing experiments, it was found that the calcium carbonate content at different depths was very low. Therefore, it can be concluded that biological soil crusts are the dominant factor inducing localized erosion resistance rather than calcium carbonate deposition effects.

#### Salt identification

**Figure 7:**
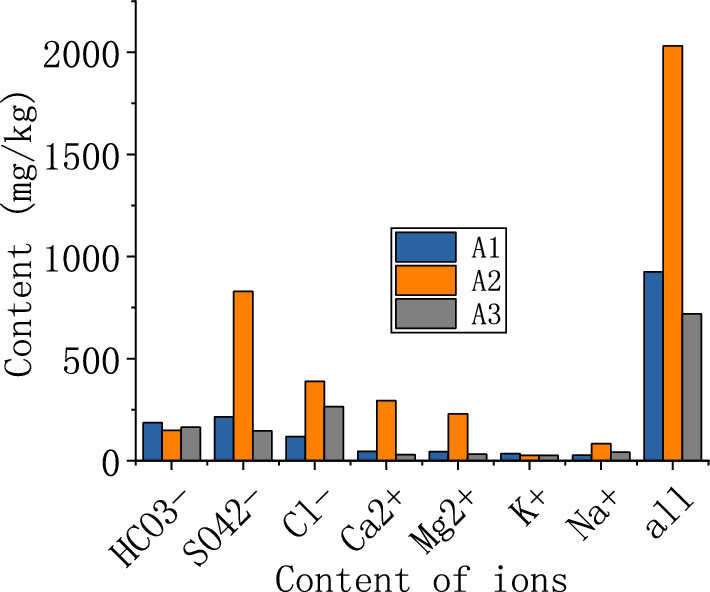
Salt concentration in the soil.

The salt content of layer A1, powder layer A2 and parent soil layer A3 were tested respectively. The test method is in accordance with the requirements of "Standards for Geotechnical Test Methods" (GB/T50123-2019), and the results are shown in Fig. 7. In terms of the total content of salts, the crust layer had the lowest, then the parent soil layer, and the pulverized layer had the highest.

#### XRD test

XRD tests can be used for qualitative and quantitative phase analysis of powder samples, which can be used to analyze the mineral composition and proportion of the layer sample A1, the layer sample A2, the wall mother soil sample A3, and the weathered rock block A4 near the wall. The test was carried out using the Smart Lab diffractometer of Nikaku, Japan, with a working environment of 40kv-200 mA and an angular speed of 2 degrees per minute.

**Figure 8:**
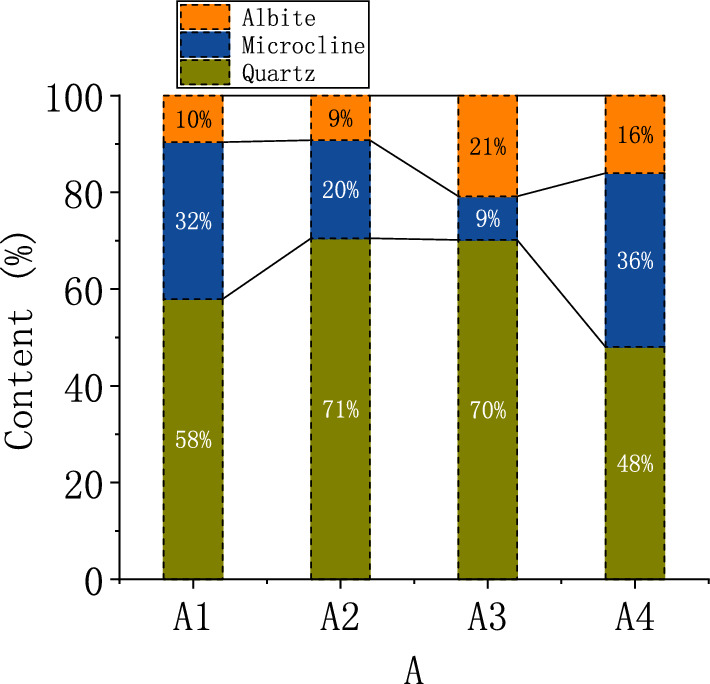
XRD test results.

The mineral composition of soil is shown in Fig. 8. As a whole, there is no significant difference in the mineral composition of the soil, mainly quartz (SiO2), microcline (K [AlSi3O8]), albite (Na2O·Al2O3·6SiO2). The composition of layer A1 mineral is basically the same as that of weathered rock A4, indicating that the three minerals with strong peak value in the diffraction pattern of layer A1 are primary minerals, while the peak value of secondary minerals is lower. The content of quartz in soil is higher than that of weathered rock, indicating that quartz is less easily decomposed by weathering in soil.

### Rain erosion test

#### Field erosion test of rammed earth Great Wall

At the rammed earth wall site, three sites were selected as experimental sites. At erosion site 1, the wall was completely covered by a gray‒green thin-layer structure, denoted as E1. At erosion site 2, part of the city wall was covered by a gray‒green thin-layer structure, denoted as E2. At erosion site 3, there was an ordinary section of wall that had not yet been covered, denoted as E3. Erosion experiments were carried out using a pressure pump with a water spray rate of 3.365 mL/min. The sprinkler was 30 cm away from the city wall surface, the pressure of the outlet pipe was 0.257 MPa, and the rain flushing time was 5 min/48 h. Plastic buckets were used to collect the washed soil and water mixture. After standing for 48 h, the water in the bucket was extracted and dried, and the quality of the washed soil was measured.

#### Laboratory erosion test

**Figure 9:**
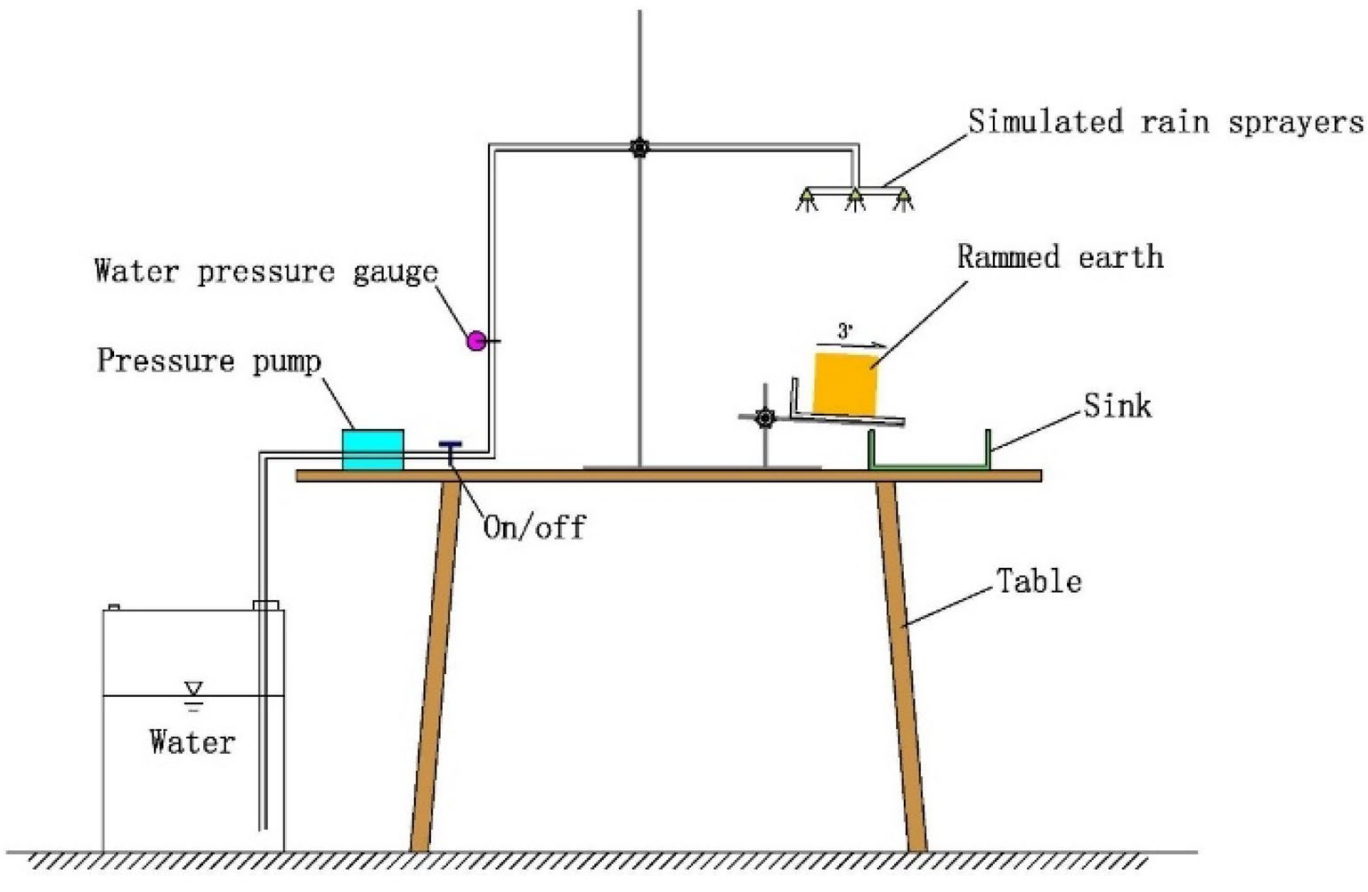
Rainfall erosion test chart.

The original sample D1 of the gray‒green thin-layer structure, the original sample D2 of the parent soil layer, and the rammed soil sample D3 made from the parent soil were used for the laboratory rainfall erosion test, which was used to compare and test the erosion resistance of the undisturbed samples of the gray‒green thin-layer structure with the parent soil and rammed soil samples. The laboratory erosion test system is shown in Fig. 9. The pressure pump was used at 3.365 mL/min. The distance between the spray port and the soil sample was 30 cm, the pressure of the outlet pipe was 0.257 MPa, and the rainfall flushing time was 5 min. The erosion process was recorded with a camera. After the erosion test, the soil‒water mixture was dried to measure the quality of the scoured soil.

### Soil crust property test

#### Immersion test

The gray‒green thin-layer structure on the erosion resistance wall surface found in the field was removed with a knife and was cultured in deionized water and BG11 medium prepared in laboratory^41^. The composition of BG11 (Culture medium for soil crust) is shown in Table 4. The surface changes were observed after 7 days.

**Table 4:** BG11 medium ratio.

Medicine	Dosage/(g/L)
NaNO_3_	1.500
K_2_HPO_4_·3H_2_O	0.040
MgSO_4_·3H_2_O	0.075
CaCl_2_·2H_2_O	0.036
Citric acid	0.006
Ferric ammonium citrate	0.006
EDTANa2	0.001
Na_2_CO_3_	0.020
H_3_BO_3_	2.860
MnCl_2_·H_2_O	1.810
ZnSO_4_·7H_2_O	0.222
CuSO_4_·5H_2_O	0.079
Na_2_MOO_4_·2H_2_O	0.390
Co(NO_3_)_2_·6H_2_O	0.049

#### Genome sequencing

**Figure 10:**
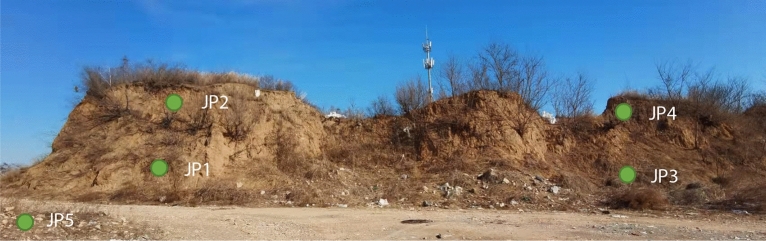
Sampling points of the crust samples of the Xiaowan Gap rammed earth Great Wall.

Genome sequencing was performed on the surface layer of 0–3 mm undisturbed gray‒green thin-layer structure samples from the upper, middle, lower and surrounding surfaces of the Great Wall. The sampling locations are shown in Table 5 and Fig. 10. The test process was DNA extraction → PCR amplification → amplicon library construction → on-machine sequencing.

**Table 5:** Sampling locations.

Number	Sampling location
JP1	Point 1, lower part of the Great Wall, 1.5 m from the ground
JP2	Point 2, upper part of the Great Wall, 1.5 m from the top of the wall
JP3	Point 3, lower part of the Great Wall, 1.5 m from the ground
JP4	Point 4, upper part of the Great Wall, 1.5 m from the top of the wall
JP5	Natural ground at the bottom of the Great Wall, 10 m away from the Great Wall

### SEM test

The gray‒green thin-layer structure sample A1 and the parent soil layer A3 were selected for drying treatment and polished into 3 mm × 3 mm × 5 mm cubes with a knife and sandpaper for SEM observation. The test instrument used was a VEGA3 (LM) fully automated tungsten filament scanning electron microscope produced by Czech TESCAN. The experimental magnification was 500–20,000 times.

## Results

### Rainfall erosion test results

#### Field erosion test results

**Figure 11:**
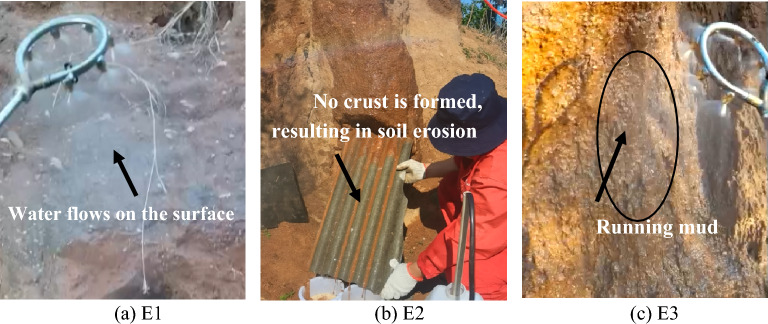
Field erosion resistance test.

The 5-min field erosion test results are shown in Fig. 11. It can be seen from the figure that for E1, after the water droplets contacted the crust surface, the water flowed along the surface of the gray‒green thin-layer structure, the water body was clear, and the crust layer surface did not change within 5 min. For E2, water infiltration occurred at the position where the crust layer was not yet formed, the phenomenon of mud flow occurred after 2 min, and the phenomenon of clod collapsing and falling occurred after 4 min. E3 had not yet formed a crust layer. After the parent soil layer was in direct contact with the water droplets, the water body infiltrated, and the phenomenon of mud flow and slumping occurred at 1 min. The soil body collapsed at 2 min, and the mud block flowed together with the water flow at 3 min, causing serious soil erosion. After 5 min of erosion, the soil mass was dried and weighed as follows: 32.77 g (E1), 895.10 g (E2), and 2926.11 g (E3). It can be seen that the gray‒green thin-layer structure in the field has good erosion resistance.

#### Laboratory erosion test results

**Figure 12:**
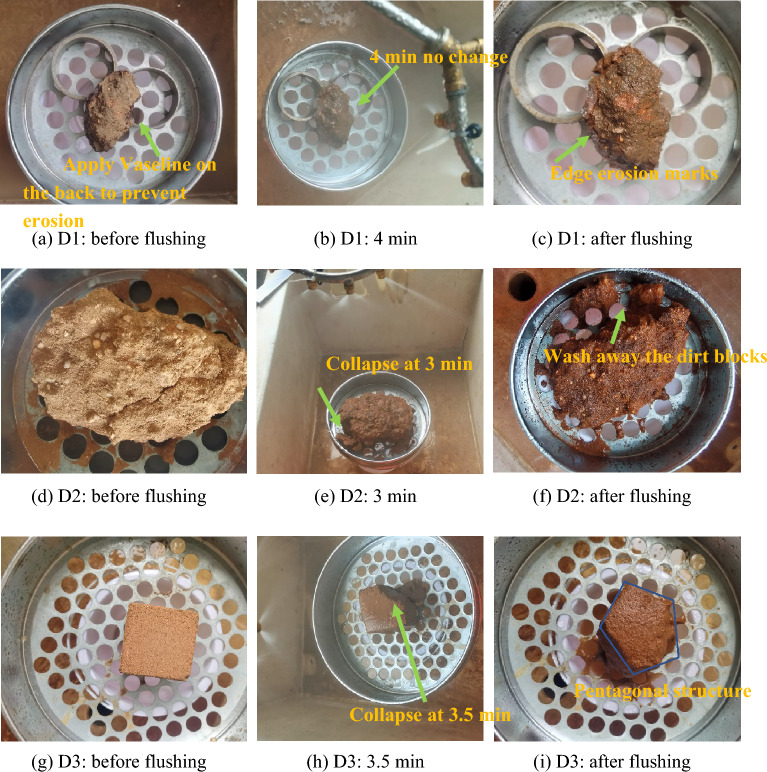
Laboratory erosion resistance test.

The laboratory 5-min erosion test results are shown in Fig. 12. As seen from the figure, the water droplets of the original sample of the D1 cortex layer flowed along the surface after encountering water, the overall stability was better, and only the erosion marks appeared at the edges. This erosion mark was caused by the untreated edge of the soil sample, and the surface was wet after washing. The undisturbed parent soil sample D2 was changed by water infiltration and wetting processes in the first 2 min after encountering water. At 3 min, the edge soil block fell, and at 3.5 min, the large block slipped and collapsed. After 4 min, the sample was washed by rain and mud flows. The rammed earth sample D3 was changed by a water infiltration process in the first 3 min after encountering water. Small gullies appeared in the local edges and corners, and the edges and corners slumped at 3.5 min. After 4 min and 10 s, the remaining edges and corners began to slump. Finally, after 5 min, all four edges and corners collapsed to form a pentagonal structure.

Before erosion, the masses of samples D1, D2, and D3 were 108.87 g, 912.65 g, and 572.62 g, respectively. After 5 min of erosion, the resulting sample masses were 103.72 g, 663.11 g, and 392.95 g, accounting for 95.27%, 72.66%, and 68.62%, respectively. The overall erosion resistance results show an order of D1 > D2 > D3. It can be seen that the gray‒green thin-layer structure also has a very good erosion resistance performance.

### Immersion test results

**Figure 13:**
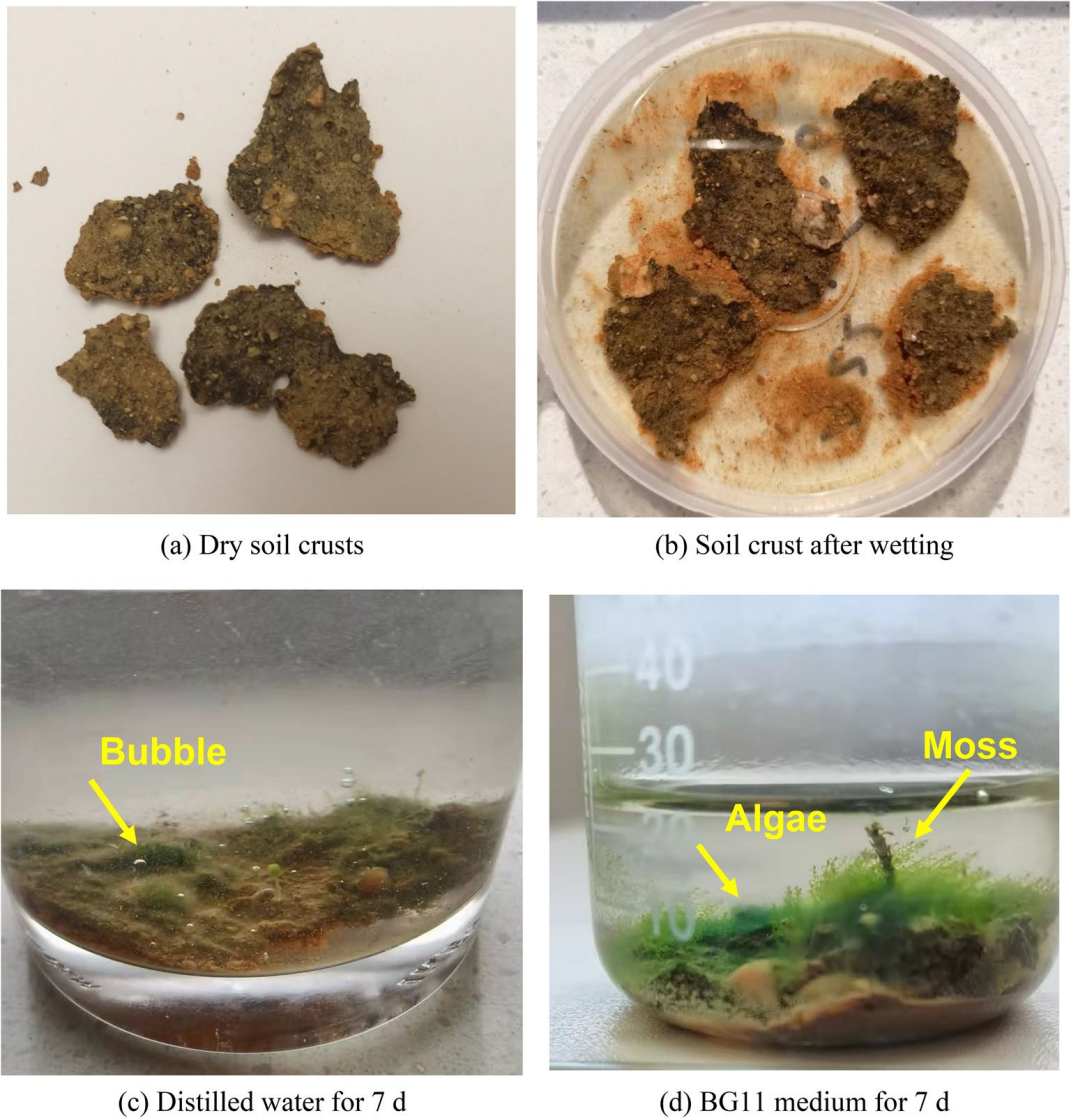
Growth status of soil crust samples after culture.

The gray‒green thin-layer structure samples (Fig. 13a) were placed in distilled water and BG11 culture medium. Figure 13b shows that the gray‒green thin-layer structure sample did not disintegrate after being wetted in water, indicating that it has a certain bond strength and disintegration resistance. It can be seen from Fig. 13c and d that after 7 d of incubating the gray‒green thin-layer structure sample, bubbles formed in the water, and biological growth occurred on the surface. The gray‒green thin-layer structure samples were biological crust, and biological crusts produce air bubbles during photosynthesis in water. Figure 13d shows that algae and moss grew on the surface of the biological crust. This shows that biological crust is a complex system that involves a process of continuous evolution and growth by the joint action of algae and moss. When water and nutrients are sufficient and light and temperature are suitable for growth, and the biological crusts will transition from algal crusts to moss crusts.

### Genome sequencing results

**Figure 14:**
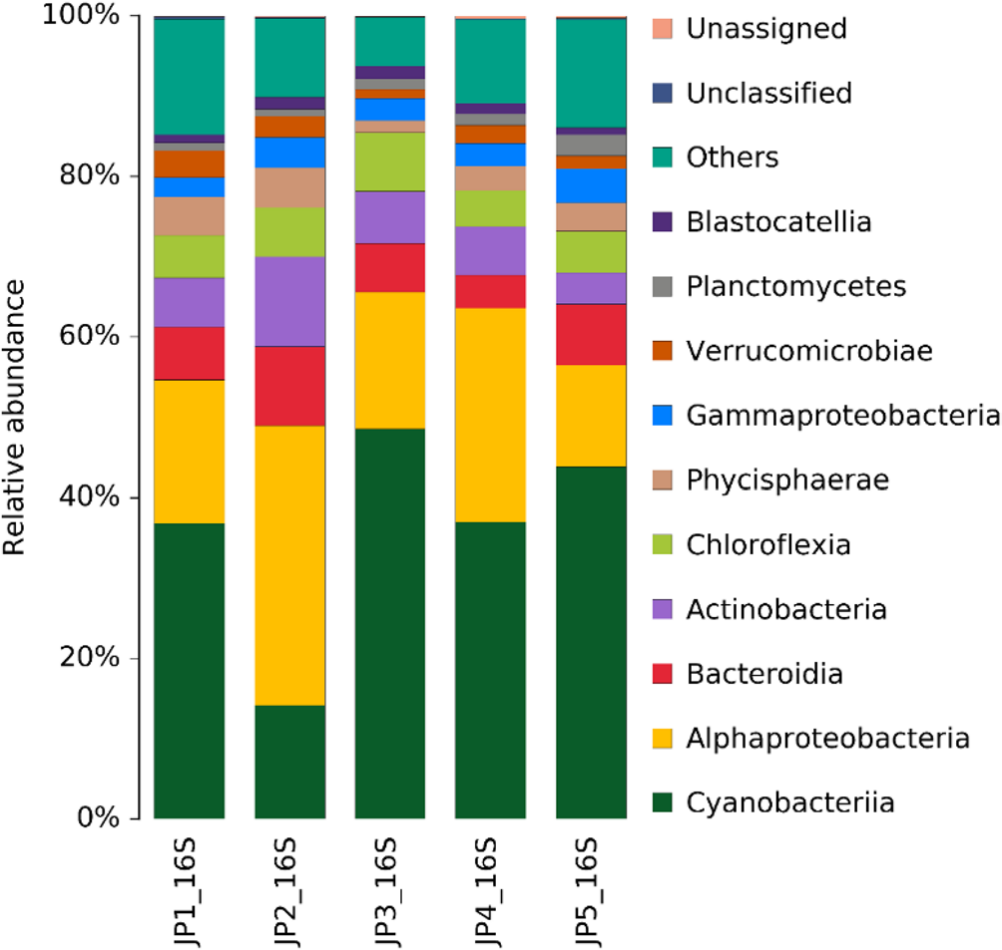
Stacked bar chart of the distribution of soil crust DNA sequencing species.

Through genome sequencing of the gray‒green thin-layer structure, it was observed that the gray‒green thin-layer structure is a biological soil crust layer. The test results are shown in Table 6. The soil crust layer of the organism contains a total of 28 phyla, 65 classes, and 140 orders, and the composition mainly comprises cyanobacteria and alphaproteobacteria, accounting for 60% (Fig. 14). This shows that the soil crust layer is a composite soil crust layer dominated by algae and supplemented by fungi.

**Table 6:** Species distribution data table of soil crust DNA sequencing.

Sample	Kingdom	Phylum	Class	Order
JP1	1	23	53	106
JP2	1	18	38	69
JP3	1	21	46	88
JP4	1	21	46	85
JP5	1	28	62	126
Total	1	28	65	140

### SEM microstructure

**Figure 15:**
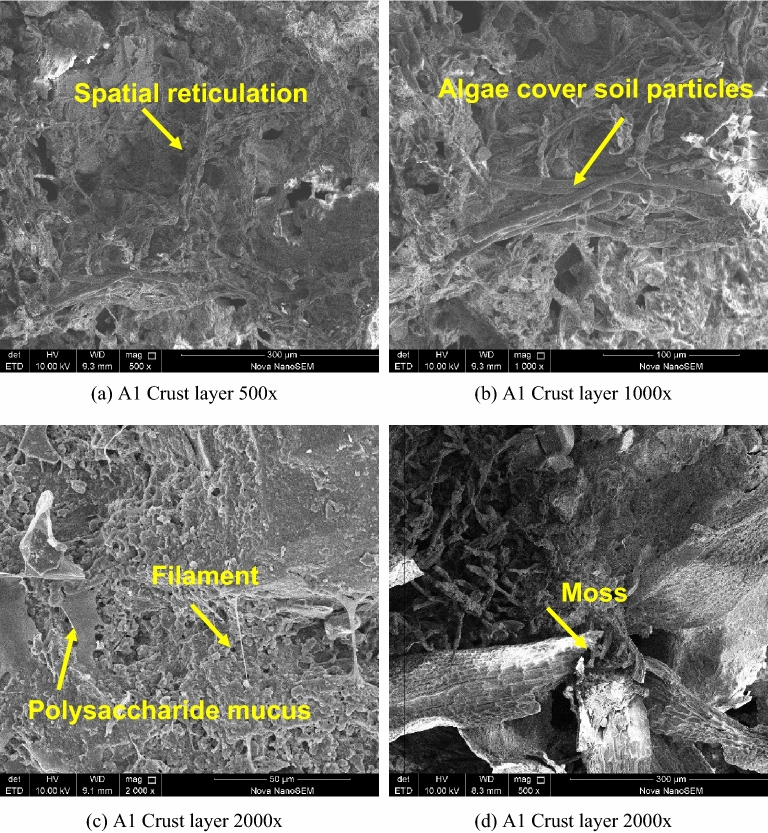
SEM photo of the crust layer.

Figure 15 shows the SEM results of A1 junction cortex with magnification of 500–2000× for comparative observation. Under the scanning electron microscope, it can be seen clearly from Fig. 15b that the algae present filamentous, overlapping with each other and covering the surface of the soil particles. As can be seen from Fig. 15c, the polysaccharide mucous secreted by algae covers the surface of the soil particles in a flake form and wants to bond with the soil particles, and mycelium can be observed in some sections. Figure 15d shows the appearance of moss, which obviously grows outside the soil particles. As can be seen from Fig. 15a, as algal filaments grow in line with each other and form overlapping bonds, they intertwine with soil particles and form a spatial network structure on the soil surface. At the same time, the network structure is covered by a large amount of polysaccharide slime, making the soil surface smoother and more delicate.

**Figure 16:**
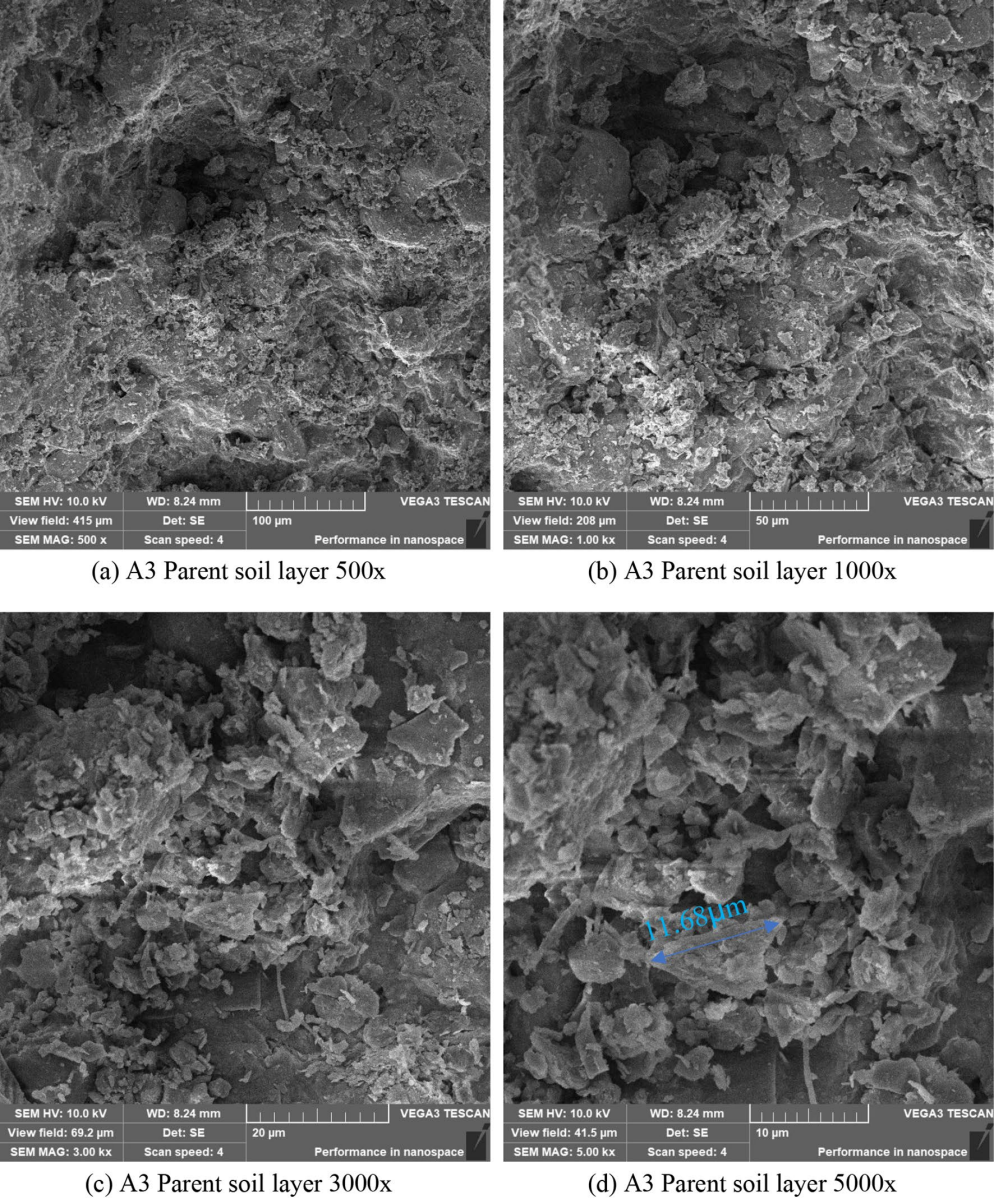
SEM photo of the parent soil layer.

Figure 16 is a scanning electron microscope image of the parent soil layer sample A3, magnification 500–5000x. It can be seen from the figure that the soil mass is in sheet structure, and the particle size of the soil mass is about 5 μm. Filamentous, spherical, rod-shaped, and soil particle bonding phenomena were not observed for the soil surface. ImageJ software was used to magnify the image in Fig. 15d by 5000× to measure the soil particle diameter. The maximum particle size of the soil particles was 11.68 μm, and the minimum particle size was 0.35 μm.

## Discussion

### Identification of erosion resistance of local rammed soil

The erosion resistance of undisturbed soil with gray-green soil crust is obviously higher than that of other soil types through field and indoor erosion experiments. Therefore, it can be determined that the local erosion resistance of the rammed earth Great Wall of Shanhaiguan is caused by the gray-green soil crust. Since most biological soil crusts are formed in arid and semi-arid desert areas^19,20^, we doubt whether it is due to calcified crusts in rammed soil or the addition of glutinous rice pulp. However, we did not find this phenomenon by testing the rammed earth properties of samples A1, A2, and A3. After the immersion experiment, we found that this soil crust could breathe and discharge CO_2_, so we judged that this soil crust was biological crust. Finally, through genome sequencing, we found that this is a composite soil crust composed mainly of cyanobacteria and supplemented by fungi. Moss was also found under scanning electron microscopy. Previous scholars believed that soil crust followed a natural evolution process, from simple to complex, from low to high, from algal crust to moss crust^21,22^.

### Erosion protection mechanism of biological crusts on the rammed earth Great Wall

**Figure 17:**
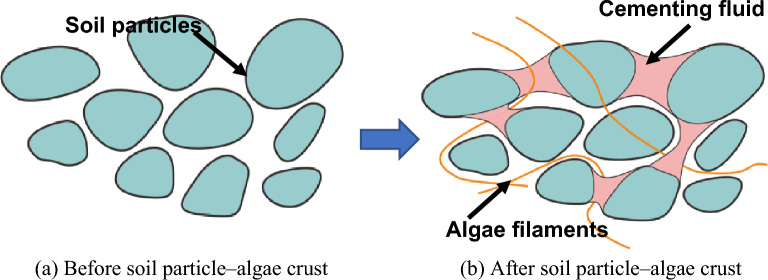
Process of algal crusting.

The experimental results show that algal biological crusts can effectively prevent rainwater erosion. The erosion resistance mechanism of algal crust is explained as follows: First, from a macroscopic perspective, algal crusts form a protective layer on the wall surface, which can effectively resist and alleviate the impact of water droplets on soil particles, reduce soil splash erosion and infiltration, and increase surface runoff^42,43^. On the other hand, after rainfall, algae can quickly absorb residual water and reduce soil damage caused by wetting. Second, from a microscopic point of view, algae secrete polysaccharides, lipids and proteins under photosynthesis that have a cohesive effect, which can effectively bind soil particles^44,45^, as shown in Fig. 17. On the other hand, algal filaments and mycelia wrap and wind among the soil particles, which can firmly bind soil particles and form a special multilayer network structure with polysaccharide mucinous secretions, thus enhancing the soil surface stability.

The findings of this study not only have applications in the protection of the Great Wall against rainwater erosion but also offer a viable method for the conservation of similar rammed earth heritage sites. Biological soil crusts, as part of the local biotic community, exhibit excellent adaptability and environmental friendliness, capable of sustaining themselves on the surfaces of rammed earth sites over the long term. This represents a distinct advantage over traditional engineering protective measures.

### Further problems to be solved

Biological crusts have a positive effect on the erosion resistance of cultural relics in soil sites, which has been confirmed by field observations and the above experiments. The mitigation mechanism for rain erosion has also been explained. However, which algae are the dominant algal species in biological crusts, how algae and fungi play a protective role together, and the inhibitory effect of algae crusts on the growth of plants on the surface of the Great Wall also require further in-depth research. Our future research endeavors involve extracting, isolating, and on-site inoculation demonstrations of the Great Wall.

The Great Wall of Shanhaiguan is adjacent to the Bohai Bay, which has a temperate continental monsoon climate and four distinct seasons. An important factor affecting its durability is the freeze–thaw process. In the future, we will carry out research on the impact of freezing and thawing on the durability of rammed earth sites.

## Conclusions

This work reveals the reason of local erosion resistance of rammed earth Great Wall site. The following conclusions are obtained from the material composition and erosion test of rammed earth:

1. The improvement of local anti-erosion ability of Xiaowan Gap Great Wall is not due to the formation of calcification and precipitation on the soil surface and the change of mineral composition in the wall. It is caused by the formation of biological soil crust on the soil surface.
2. The polysaccharides produced by the algae-dominated biological crusts formed on the soil surface of the Great Wall played a role in cementing the soil particles. The algal hyphae, mycelium and cementing liquid together form a new spatial network structure, which improves the soil erosion resistance.
3. Hydroponics and genome sequencing revealed various organisms in the biological crust, including different algae, mosses and fungi. Which type of algae is more beneficial for soil reinforcement and which type of algae can be used to reproduce and cultivate the dominant algae for cultural relic protection are worth future discussion.

## Data Availability

All data included in this study are available upon request by contact with the corresponding author. The datasets generated and/or analysed during the current study are not publicly available due to the confidentiality of project achievements but are available from the corresponding author on reasonable request.
